# Cynomolgus monkey’s retina volume reference database based on hybrid deep learning optical coherence tomography segmentation

**DOI:** 10.1038/s41598-023-32739-6

**Published:** 2023-04-09

**Authors:** Nora Denk, Christian Freichel, Philippe Valmaggia, Nadja Inglin, Hendrik P. N. Scholl, Pascal Kaiser, Sylvie Wise, Marc Vezina, Peter M. Maloca

**Affiliations:** 1 Pharma Research and Early Development (pRED), Pharmaceutical Sciences (PS), Roche, Innovation Center Basel, 4070 Basel, Switzerland; 2 Department of Ophthalmology, University Hospital Basel, 4031 Basel, Switzerland; 3 Institute of Molecular and Clinical Ophthalmology Basel, 4031 Basel, Switzerland; 4 Supercomputing Systems, 8005 Zurich, Switzerland; 5 Charles River Laboratories, Senneville, QC H9X 1C1 Canada; 6 Moorfields Eye Hospital NHS Foundation Trust, London, EC1V 2PD UK

**Keywords:** Retina, Imaging and sensing, Preclinical research

## Abstract

Cynomolgus monkeys (*Macaca fascicularis*) are commonly used in pre-clinical ocular studies. However, studies that report the morphological features of the macaque retina are based only on minimal sample sizes; therefore, little is known about the normal distribution and background variation. This study was conducted using optical coherence tomography (OCT) imaging to investigate the variations in retinal volumes of healthy cynomolgus monkeys and the effects of sex, origin, and eye side on the retinal volumes to establish a comprehensive reference database. A machine-learning algorithm was employed to segment the retina within the OCT data (i.e., generated pixel-wise labels). Furthermore, a classical computer vision algorithm has identified the deepest point in a foveolar depression. The retinal volumes were determined and analyzed based on this reference point and segmented retinal compartments. Notably, the overall foveolar mean volume in zone 1, which is the region of the sharpest vision, was 0.205 mm^3^ (range 0.154–0.268 mm^3^), with a relatively low coefficient of variation of 7.9%. Generally, retinal volumes exhibit a relatively low degree of variation. However, significant differences in the retinal volumes due to the monkey’s origin were identified. Additionally, sex had a significant impact on the paracentral retinal volume. Therefore, the origin and sex of cynomolgus monkeys should be considered when evaluating the macaque retinal volumes based on this dataset.

## Introduction

Because cynomolgus monkeys (*Macaca fascicularis*) have close morphological ocular similarity to humans, they are commonly used in preclinical research. Notably, the presence of the fovea^1^, which is the site of best visual acuity, is one of the main reasons for using non-human primates (NHPs) in ocular studies. Retinal morphology, which includes the fovea, is commonly assessed using optical coherence tomography (OCT) in the preclinical settings^2–5^. Numerous examples include disease models and safety evaluations. In a cynomolgus monkey model of Parkinson’s disease, the average retinal nerve fiber thickness assessed via OCT was lower than that of the controls^6^. Bantseev et al.^7^ determined the no-observable effect level of endotoxins in cynomolgus monkeys using OCT assessment. Additionally, longitudinal studies have demonstrated that retinal changes occur with increasing age^8^ and that elevated intraocular pressure is associated with decreased retinal nerve fiber layer thickness^9^.

Despite the general morphological similarities between cynomolgus monkeys and humans, there are significant natural variations in foveal contours even within identical species when using two-dimensional OCT retinal measurements of the fovea^10^. Therefore, the animals’ geographic origin may play a crucial role in neuroretinal development. Moreover, knowledge of these natural variations in retinal morphology, such as retinal thickness and vessel parameters^11,12^, is vital when planning the study design and assessing retinal thickness measurements from cynomolgus monkey’s retina to prevent misinterpreting the natural variations with suspected retinal pathology.

Another challenge when applying OCT readouts in the preclinical setting is a localization issue. Compared with humans, cynomolgus monkeys cannot follow an operator’s instructions to maintain a particular fixation target during imaging. Therefore, the positioning of the OCT scan depends on the examiner’s judgment and expertise. Displacement of scans can significantly impact readouts. For example, when measuring the optic disc, the circular scan displacement causes iatrogenic deviations of the circumpapillary retinal nerve fiber layer thickness, which increases as the center offset increases^13^. Therefore, automatic methods were used in this study to prevent such artificial deviations by determining the deepest point in the (healthy) fovea as a landmark for further image analysis^10^.

This study was conducted to provide a reference database for OCT cynomolgus monkey retinal volumes, which depend on the origin, sex, and eye laterality. Moreover, this study compares the results obtained for the retinal volumes to results obtained for the choroidal volumes, which are based on the same OCT scans of the same cynomolgus monkeys and were published in a previous choroid study^11^.

## Materials and methods

### Animals and husbandry

This study used preexisting, retrospective data that were initially derived from routine investigations conducted during pharmaceutical product development. Therefore, no additional animal experimentation was performed in this study. Specifically OCT scans of ocular safety studies obtained from treatment-naïve cynomolgus monkeys (*Macaca fascicularis*) of both sexes were retrospectively analyzed in this study. The original safety studies were reviewed and approved by the Institutional Animal Care and Use Committees (IACUC) of the respective institutions (Charles River Laboratories Montreal, ULC IACUC (CR-MTL IACUC), IACUC Charles River Laboratories Reno (OLAW Assurance No. D16-00594), and IACUC (Covance Laboratories Inc., Madison, WI, USA) [OLAW Assurance #D16-00137 (A3218-01)]. The study is reported in accordance with ARRIVE guidelines. All animals were handled and used strictly following the guidelines of the US National Research Council or Canadian Council on Animal Care. These animals were bred specifically for laboratory use and obtained from certified suppliers in two geographic regions: Mauritius and Asia. The animals were group-housed in stainless steel cages according to the European housing standards described in Annex III of Directive 2010/63/EU. The rooms were maintained at a constant temperature of 20–26 °C, with humidity between 20 and 70%, and a light–dark cycle of 12:12 h was induced. The diet comprised standard pellets augmented with fresh fruits and vegetables. Municipal tap water, which was treated with reverse osmosis and ultraviolet (UV) irradiation, was freely available to each animal through an automated watering system. The animals were also provided psychological and environmental enrichment, except during the study procedures and activities.

### OCT image acquisition

Imaging was performed under general anesthesia (ketamine, 10 mg/kg intramuscularly (IM); dexmedetomidine, 25 µg/kg IM) to minimize stress for the animals and ensure a stable eye position. Immediately before the start of OCT imaging, a single dose of midazolam (0.2 mg/kg IM) was administered to keep the eyes centrally positioned. The pupils were dilated using a topical administration of tropicamide prior imaging. The OCT data were obtained using a spectral-domain OCT device (Heidelberg Engineering, Heidelberg, Germany). Horizontal OCT scan lines had a size of 20°, and 25 raster lines were applied (spacing, 221 μm; scan length 5.3 mm, 512 × 496 pixels; scan depth, 1.9 mm). OCT data were exported from the device in a bitmap image data format (BMP).

### Measuring retinal volumes

**Figure 1:**
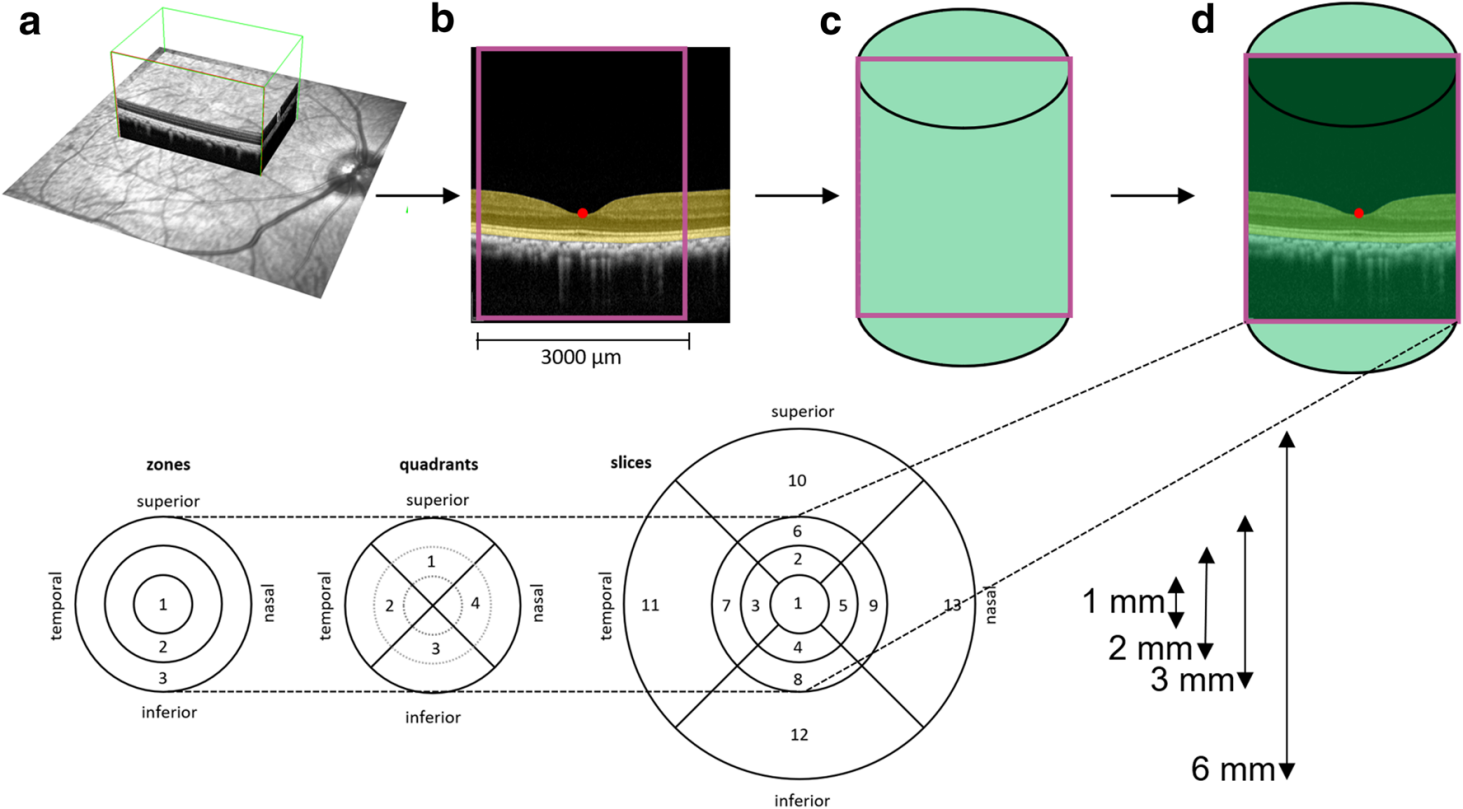
Visualization of the optical coherence tomography (OCT) retinal volume measurements. (**a**) A central retinal OCT volume was acquired (depicted with the green box). (**b**) In the first analysis step, the retina (highlighted in yellow) was separated from the rest of the tissue using a validated deep learning algorithm. In the second step, a classical algorithm automatically detects the deepest point within the fovea (marked as a red dot and labelled nulla). A field of interest with a diameter of 3000 µm was defined starting from nulla (illustrated with the purple rectangle). (**c**) And rotated within the OCT volume (symbolized as a green cylinder). (**d**) This enabled the volumetric measurements in ring areas (1, 2, and 3 mm) of four quadrants and slices, as illustrated.

The first two steps of the image processing pipeline were described previously^10^. In summary, in the first step, the retina was segmented on all B-scans of the OCT scans using a deep learning-based, semantic image segmentation algorithm (Fig. 1). The algorithm is based on a modified U-Net architecture and was described in detail and validated previously^12^. Basically, the algorithm assigns one of the labels vitreous, retina, choroid, and sclera to each pixel of a B-scan. In the second step, an algorithm based on classical computer vision was applied to detect the deepest location automatically within the foveolar depression (termed nulla) in each OCT volume. The nulla is defined as the deepest point of the inner limiting membrane (ILM), which separates the vitreous from the retina (Fig. 1). The ILM was identified based on the semantic segmentation maps that were generated by the deep learning-based algorithm. The algorithm used for the nulla-finding was introduced and described in detail in a previous study^10^. The nulla is particularly important because it determines the center of the fovea, which is responsible for sharp central vision. Finally, according to the position of the nulla, a two-dimensional rectangular region of interest (ROI) was placed in the B-scan plane with a total width of 3000 µm and the nulla at its center, which defined the longitudinal section of a cylindrical region (Fig. 1). Furthermore, this cylindrical region was used to define the retinal volumetric regions. As illustrated in Fig. 1, this study determined the retinal volumes of three concentric zones (Z1–Z3), four quadrants (Q1–Q4), and nine slices (S1–S9).The semantic segmentation maps, which were generated by the deep learning-based algorithm, were used to count the voxels in each of the zones, quadrants, and slices. From these voxel counts, finally, the respective volumes were determined. Note that zone 1 and slice 1 represent the same volume. This labelling was done in order to be able to use a specific and distinctive terminology for sub-analyses. Therefore, slice 1 was omitted in the results section, and only zone 1 is listed.

### Statistical analysis

Summary statistics, which include mean, standard deviation, median, minimum, maximum, and coefficient of variation (CV), were calculated for each measured volume for subgroups of the data (e.g., for females of Asian origin). Additionally, overall summary statistics based on all eyes were calculated for zone 1 since it is the region of sharp central vision.

Pearson correlation analysis was performed to investigate the correlation between Z1–Z3, Q1–Q4, and S1–S9. Moreover, principal component analysis (PCA) was conducted to survey the patterns of variability in S1–S9. The PCA was performed to identify latent “factors,” which can be used to explain the variability in the data.

Multivariate analysis of variance (MANOVA) was performed to test the effect of the independent variables “sex,” “origin,” and their combined interactions on the nine dependent variables S1–S9. Significance was calculated using the F statistic, which is part of the MANOVA implementation contained in the Python library statsmodels. Sixteen, eyes of unknown origin were excluded from the MANOVA procedures.

Furthermore, nine individual analyses of variance (ANOVA) were performed to examine the effect of the independent variables “sex,” “origin,” and their interaction on each of the nine dependent variables S1–S9. Sixteen eyes of unknown origin were excluded from the ANOVA procedures. ANOVA and MANOVA were performed using Python library statsmodels v0.12.1. For ANOVA, the significance of differences between the group means was calculated using the F statistic, which is contained in the ANOVA implementation of the statsmodels library. Additionally, Bonferroni correction was performed to counteract the multiple testing problem by dividing the significance levels by nine (number of individual ANOVA procedures). Variables that showed significance at *p* < 0.001/9 were indicated with ‘***’, at *p* < 0.01/9 with ‘**’, and at *p* < 0.05/9 with ‘*’.

Boxplots were used to visualize the data distribution and to show group-wise comparisons (e.g., Mauritian versus Asian origin). Boxplots were plotted with the Python library seaborn v0.11.1. All the calculations were performed using Python v3.8.5.

## Results

### General results

In total, 374 volumetric OCT recordings were obtained from 374 eyes of 203 animals. Females comprised 147 eyes (39.30%) and males 227 eyes (60.70%). Overall, 186 and 188 eyes were left (49.73%) and right (50.27%), respectively. Mauritian and Asian Monkeys also contributed 199 (53.20%) and 159 (46.80%) eyes, respectively. However, 16 eyes were of unknown origin. Furthermore, animal age and weight ranged from 30 to 50 months and 2.5 to 5.5 kg, respectively.

### Summary statistics

An overall analysis that included all 374 eyes revealed a mean retinal volume of 0.205 mm^3^ (range 0.154–0.268 mm^3^; CV, 7.9%) for zone 1, which is the region of sharp central vision. A subgroup analysis, which categorized the animals according to sex and origin, revealed a similar degree of variation in subgroups (Tables 1, 2 and 3, measured by CV).

**Table 1:** Summary statistics of retinal zone volumes regarding origin and sex.

	Stats	Zone 1					Zone 2					Zone 3				
		All	m/M	m/A	f/M	f/A	All	m/M	m/A	f/M	f/A	All	m/M	m/A	f/M	f/A
OD	Count	188	62	45	34	39	188	62	45	34	39	188	62	45	34	39
	Mean	0.205	0.200	0.213	0.199	0.207	0.768	0.767	0.792	0.752	0.754	1.375	1.375	1.383	1.369	1.367
	std	0.017	0.013	0.022	0.012	0.015	0.035	0.031	0.036	0.028	0.032	0.055	0.054	0.061	0.046	0.054
	Min	0.160	0.167	0.160	0.167	0.179	0.676	0.702	0.706	0.692	0.676	1.256	1.267	1.265	1.265	1.256
	Median	0.204	0.200	0.213	0.199	0.205	0.767	0.767	0.786	0.755	0.759	1.375	1.376	1.378	1.372	1.372
	Max	0.264	0.226	0.264	0.221	0.238	0.873	0.828	0.873	0.800	0.808	1.538	1.512	1.538	1.461	1.490
	CV	0.081	0.067	0.101	0.061	0.070	0.046	0.040	0.045	0.036	0.042	0.040	0.039	0.043	0.033	0.039
OS	Count	186	65	39	38	36	186	65	39	38	36	186	65	39	38	36
	Mean	0.205	0.202	0.214	0.200	0.207	0.770	0.771	0.795	0.755	0.753	1.378	1.376	1.383	1.374	1.376
	std	0.016	0.012	0.022	0.012	0.014	0.035	0.030	0.040	0.028	0.031	0.053	0.050	0.061	0.050	0.055
	Min	0.154	0.171	0.154	0.165	0.182	0.680	0.702	0.684	0.703	0.680	1.265	1.268	1.265	1.269	1.275
	Median	0.205	0.202	0.215	0.200	0.208	0.770	0.769	0.787	0.765	0.749	1.380	1.373	1.382	1.380	1.379
	Max	0.268	0.228	0.268	0.223	0.239	0.883	0.821	0.883	0.801	0.813	1.509	1.499	1.509	1.476	1.492
	CV	0.076	0.059	0.100	0.060	0.065	0.045	0.039	0.050	0.036	0.040	0.039	0.036	0.043	0.036	0.039

*OD* oculus dexter, *OS* oculus sinister, *Stats* statistic, *std* standard deviation, *min* minimum, *max* maximum, *CV* coefficient of variation, *m* male, *f* female, *M* Mauritius, *A* Asian, values in mm^3^. Note that slice 1 is identical to zone 1.

**Table 2:** Summary statistics of retinal quadrant volumes regarding origin and sex.

	Stats	Quadrant 1					Quadrant 2					Quadrant 3					Quadrant 4				
		All	m/M	m/A	f/M	f/A	All	m/M	m/A	f/M	f/A	All	m/M	m/A	f/M	f/A	All	m/M	m/A	f/M	f/A
OD	Count	188	62	45	34	39	188	62	45	34	39	188	62	45	34	39	188	62	45	34	39
	Mean	0.593	0.591	0.598	0.589	0.591	0.597	0.597	0.607	0.590	0.589	0.591	0.588	0.602	0.584	0.585	0.567	0.566	0.580	0.557	0.563
	std	0.024	0.024	0.026	0.021	0.022	0.024	0.023	0.027	0.018	0.023	0.025	0.023	0.028	0.021	0.025	0.021	0.019	0.023	0.017	0.018
	Min	0.544	0.545	0.551	0.544	0.547	0.535	0.555	0.535	0.544	0.535	0.534	0.542	0.547	0.547	0.534	0.521	0.535	0.526	0.521	0.524
	Median	0.593	0.589	0.596	0.587	0.593	0.595	0.595	0.605	0.588	0.590	0.590	0.586	0.599	0.585	0.588	0.567	0.564	0.580	0.558	0.564
	Max	0.675	0.639	0.675	0.628	0.635	0.682	0.651	0.682	0.628	0.626	0.680	0.649	0.680	0.632	0.635	0.635	0.606	0.635	0.601	0.602
	CV	0.040	0.041	0.043	0.034	0.036	0.040	0.038	0.044	0.030	0.039	0.043	0.039	0.046	0.035	0.042	0.037	0.033	0.039	0.031	0.032
OS	Count	186	65	39	38	36	186	65	39	38	36	186	65	39	38	36	186	65	39	38	36
	Mean	0.594	0.592	0.598	0.591	0.592	0.600	0.600	0.610	0.594	0.591	0.592	0.589	0.603	0.586	0.590	0.567	0.567	0.580	0.558	0.562
	std	0.024	0.021	0.030	0.021	0.022	0.023	0.020	0.029	0.019	0.022	0.025	0.023	0.029	0.022	0.022	0.021	0.018	0.023	0.019	0.018
	Min	0.517	0.549	0.517	0.551	0.550	0.533	0.559	0.533	0.556	0.544	0.539	0.543	0.539	0.550	0.539	0.517	0.532	0.519	0.517	0.527
	Median	0.593	0.591	0.595	0.593	0.593	0.599	0.599	0.609	0.594	0.591	0.591	0.589	0.599	0.583	0.589	0.567	0.567	0.581	0.558	0.562
	Max	0.672	0.644	0.672	0.634	0.635	0.684	0.644	0.684	0.632	0.628	0.663	0.642	0.663	0.632	0.633	0.634	0.602	0.634	0.606	0.607
	CV	0.040	0.036	0.050	0.035	0.036	0.038	0.033	0.046	0.032	0.037	0.041	0.038	0.048	0.037	0.038	0.037	0.032	0.040	0.034	0.032

*OD* oculus dexter, *OS* oculus sinister, *Stats* statistics, *std* standard deviation, *min* minimum, *max* maximum, *CV* coefficient of variation, *m* male, *f* female, *M* Mauritius, *A* Asian, values in mm^3^. Note that slice 1 is identical to zone 1.

**Table 3:** Summary statistics of retinal slice volume regarding origin and sex.

	Stats	Slice 2					Slice 3					Slice 4					Slice 5				
		All	m/M	m/A	f/M	f/A	All	m/M	m/A	f/M	f/A	All	m/M	m/A	f/M	f/A	All	m/M	m/A	f/M	f/A
OD	Count	188	62	45	34	39	188	62	45	34	39	188	62	45	34	39	188	62	45	34	39
	Mean	0.191	0.192	0.199	0.185	0.185	0.199	0.198	0.201	0.198	0.197	0.188	0.188	0.198	0.181	0.181	0.191	0.189	0.194	0.189	0.191
	std	0.012	0.011	0.011	0.009	0.010	0.010	0.009	0.012	0.007	0.009	0.012	0.009	0.012	0.009	0.010	0.009	0.008	0.010	0.007	0.008
	Min	0.163	0.165	0.179	0.169	0.163	0.171	0.175	0.171	0.180	0.179	0.151	0.170	0.179	0.163	0.151	0.170	0.175	0.170	0.176	0.176
	Median	0.190	0.191	0.201	0.186	0.186	0.199	0.198	0.200	0.198	0.199	0.186	0.188	0.200	0.182	0.182	0.191	0.189	0.195	0.189	0.191
	Max	0.227	0.217	0.227	0.199	0.211	0.232	0.220	0.232	0.211	0.211	0.230	0.210	0.230	0.199	0.201	0.217	0.209	0.214	0.206	0.205
	CV	0.061	0.059	0.056	0.048	0.052	0.048	0.047	0.057	0.036	0.046	0.063	0.047	0.059	0.049	0.052	0.045	0.043	0.049	0.035	0.041
OS	Count	186	65	39	38	36	186	65	39	38	36	186	65	39	38	36	186	65	39	38	36
	Mean	0.190	0.192	0.199	0.185	0.184	0.200	0.200	0.202	0.200	0.198	0.188	0.189	0.198	0.182	0.181	0.191	0.190	0.195	0.189	0.190
	std	0.012	0.011	0.013	0.008	0.010	0.009	0.008	0.012	0.008	0.009	0.012	0.010	0.012	0.010	0.010	0.008	0.008	0.010	0.007	0.008
	Min	0.159	0.168	0.166	0.165	0.159	0.169	0.183	0.169	0.184	0.180	0.158	0.165	0.177	0.165	0.158	0.168	0.176	0.168	0.172	0.176
	Median	0.189	0.194	0.199	0.185	0.183	0.200	0.199	0.203	0.200	0.198	0.188	0.190	0.198	0.184	0.181	0.191	0.189	0.195	0.189	0.190
	Max	0.232	0.216	0.232	0.199	0.206	0.233	0.217	0.233	0.215	0.213	0.234	0.207	0.234	0.201	0.202	0.215	0.207	0.212	0.205	0.206
	CV	0.063	0.056	0.066	0.045	0.053	0.046	0.040	0.059	0.038	0.044	0.063	0.052	0.060	0.052	0.053	0.044	0.040	0.051	0.038	0.040

	Stats	Slice 6					Slice 7					Slice 8					Slice 9				
		All	m/M	m/A	f/M	f/A	All	m/M	m/A	f/M	f/A	All	m/M	m/A	f/M	f/A	All	m/M	m/A	f/M	f/A
OD	Count	188	62	45	34	39	188	62	45	34	39	188	62	45	34	39	188	62	45	34	39
	Mean	0.351	0.348	0.348	0.353	0.352	0.347	0.349	0.352	0.343	0.341	0.352	0.350	0.351	0.354	0.353	0.325	0.327	0.331	0.319	0.321
	std	0.019	0.020	0.020	0.016	0.018	0.013	0.012	0.015	0.011	0.013	0.020	0.021	0.020	0.015	0.019	0.012	0.010	0.014	0.011	0.011
	Min	0.306	0.313	0.306	0.308	0.319	0.311	0.327	0.317	0.318	0.311	0.312	0.312	0.316	0.316	0.318	0.291	0.311	0.304	0.291	0.297
	Median	0.352	0.350	0.347	0.355	0.353	0.346	0.347	0.352	0.341	0.341	0.354	0.353	0.348	0.356	0.357	0.324	0.327	0.330	0.319	0.321
	Max	0.396	0.388	0.395	0.383	0.390	0.388	0.381	0.388	0.369	0.369	0.406	0.399	0.406	0.379	0.393	0.372	0.351	0.372	0.342	0.345
	CV	0.054	0.057	0.056	0.045	0.051	0.039	0.035	0.042	0.032	0.038	0.055	0.060	0.057	0.043	0.054	0.038	0.031	0.043	0.033	0.032
OS	Count	186	65	39	38	36	186	65	39	38	36	186	65	39	38	36	186	65	39	38	36
	Mean	0.352	0.349	0.347	0.356	0.356	0.348	0.350	0.352	0.344	0.342	0.353	0.349	0.351	0.355	0.356	0.326	0.328	0.332	0.320	0.322
	std	0.019	0.019	0.020	0.018	0.018	0.013	0.011	0.015	0.011	0.013	0.020	0.020	0.021	0.017	0.019	0.012	0.010	0.013	0.011	0.010
	Min	0.308	0.316	0.312	0.308	0.321	0.314	0.327	0.315	0.319	0.314	0.310	0.310	0.310	0.316	0.320	0.288	0.311	0.301	0.288	0.296
	Median	0.354	0.348	0.345	0.359	0.360	0.347	0.348	0.354	0.343	0.341	0.353	0.347	0.347	0.359	0.358	0.325	0.328	0.330	0.320	0.323
	Max	0.391	0.388	0.388	0.387	0.391	0.387	0.374	0.387	0.368	0.370	0.397	0.395	0.393	0.387	0.393	0.370	0.349	0.370	0.346	0.346
	CV	0.055	0.054	0.058	0.049	0.051	0.037	0.031	0.042	0.031	0.038	0.055	0.055	0.059	0.048	0.052	0.035	0.030	0.039	0.034	0.032

*OD* oculus dexter, *OS* oculus sinister, *Stats* statistic, *std* standard deviation, *min* minimum, *max* maximum, *CV* coefficient of variation, *m* male, *f* female, *M* Mauritius, *A* Asian, values in mm^3^. Note that slice 1 is identical to zone 1.

**Figure 2:**
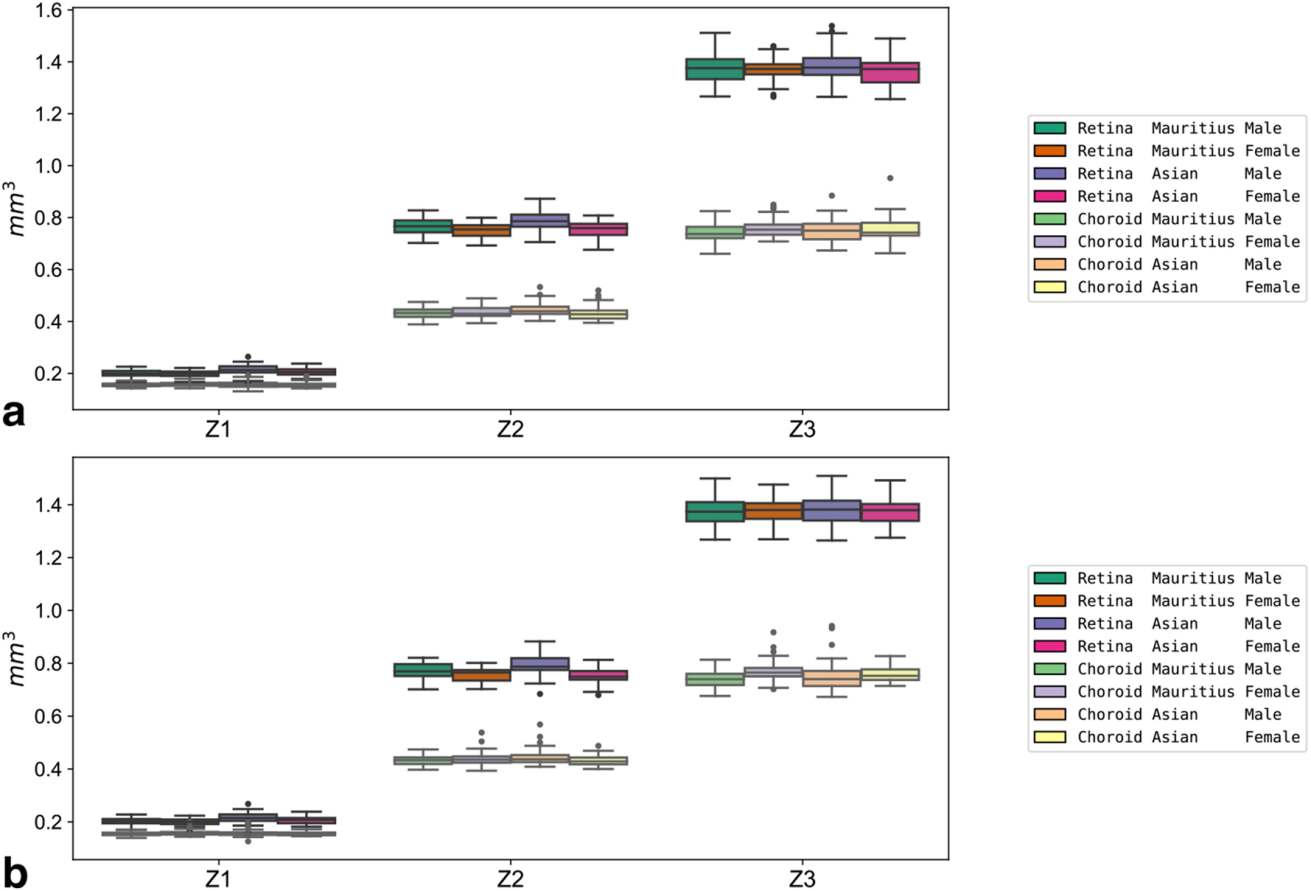
Boxplots of zone volumes. Boxplots of sex- and origin-specific variations in retinal and choroidal zone volumes for the right (**a**) and left eyes (**b**).

**Figure 3:**
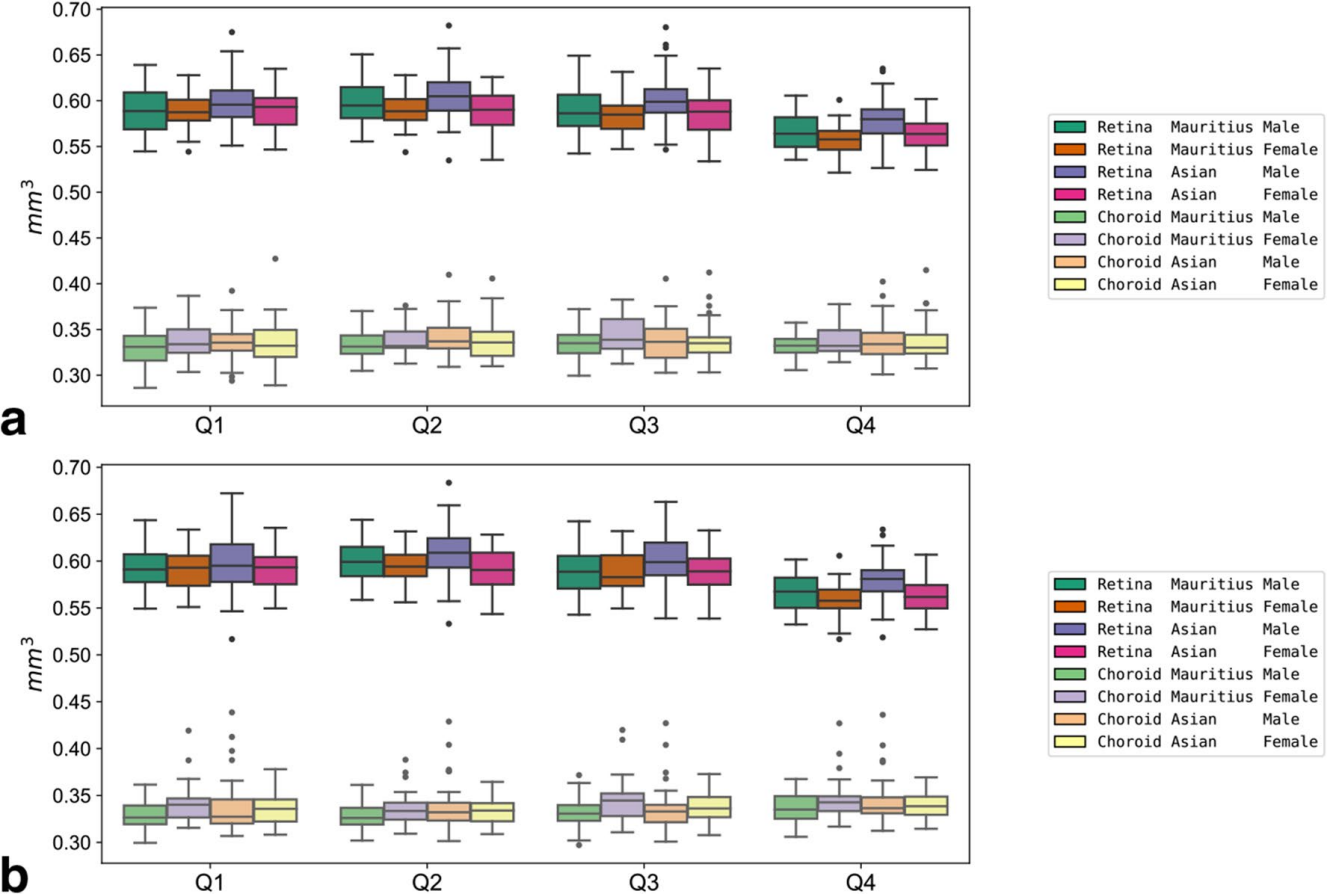
Boxplots of quadrant volumes. Boxplots of sex- and origin-specific variations in retinal and choroidal quadrant volumes for the right (**a**) and left eyes (**b**).

**Figure 4:**
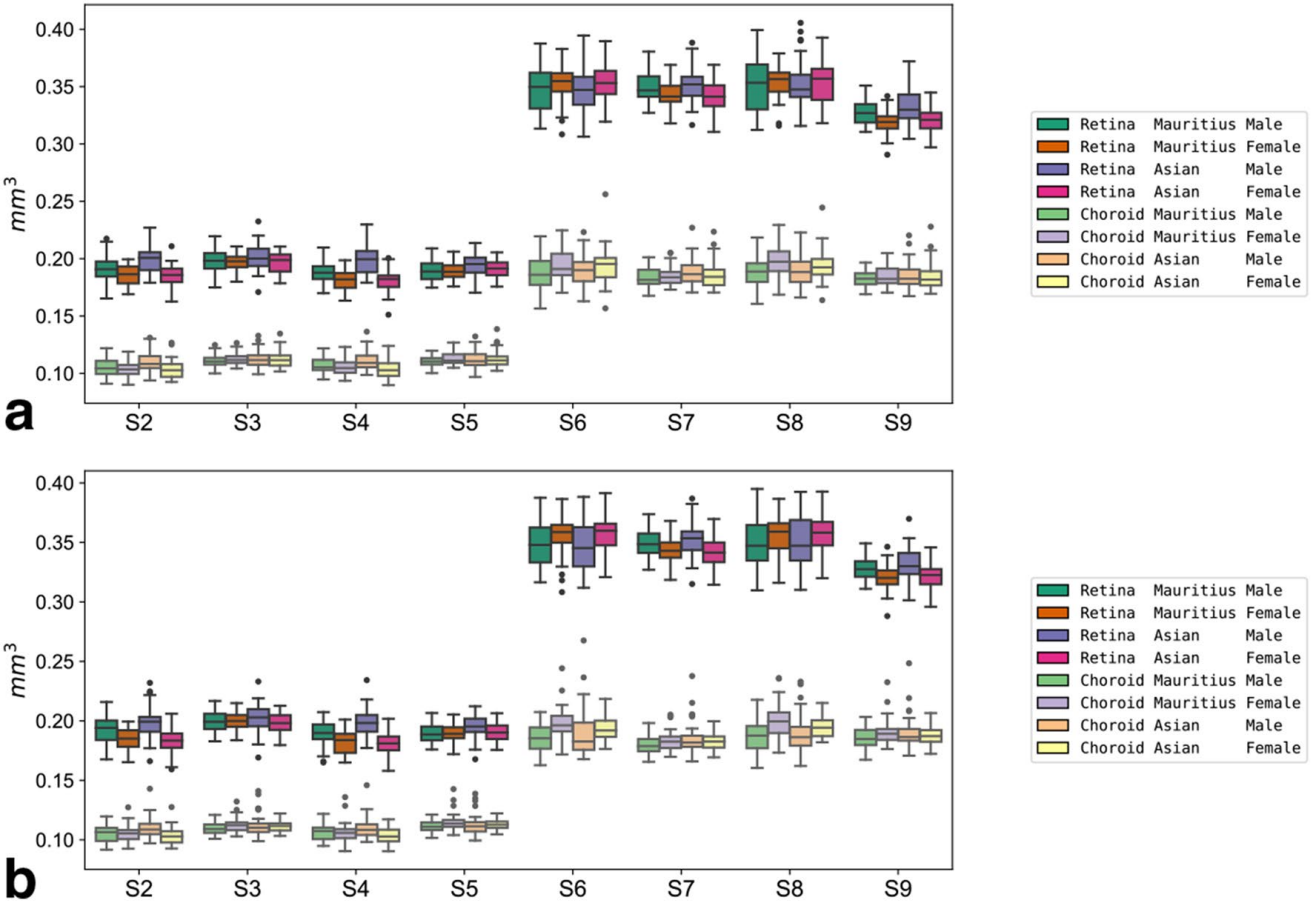
Boxplots of slice volumes. Boxplots of sex- and origin-specific variations in retinal and choroidal slice volumes for the right (**a**) and left eyes (**b**).

The distribution of the retinal volumes is visualized as boxplots in Figs. 2, 3 and 4. For ease of comparison, the boxplots also contain the data of the corresponding choroidal volumes, which was obtained in a previous study from the same eyes of the same individuals^11^. The results are depicted in the following figures:

These diagrams illustrate the relatively distinct difference in retinal volumes for slices S7, S9, quadrant Q4, and the central areas like Z1, slices S2, S4, for example. For reference, alle retinal volumes of the current study are contained in Supplementary Table S1 retina 3D.

### Correlation analysis

Table 4 presents the results of the Pearson correlation analysis, indicating that the mean correlation among zones, quadrants, and slices is 0.51 (0.35–0.69), 0.85 (0.82–0.90), and 0.49 (− 0.16 to 0.95), respectively. Additionally, the zone and quadrant coefficients are mostly composed of the nine slice coefficients (Fig. 1). Therefore, to maintain minimum statistical hypothesis tests and counteract the multiple testing problem, only the nine slice coefficients S1–S9 were used in further statistical analyses.

**Table 4:** Pearson correlation among the three zones, four quadrants, and nine slice coefficients.

Stats	Correlation					
	Among zones	Among quadrants	Among slices	zones and quadrants	Zones and slices	Quadrants and slices
Mean	0.51	0.85	0.49	0.75	0.60	0.69
Min	0.35	0.82	-0.16	0.61	0.16	0.36
25%	0.38	0.82	0.43	0.66	0.34	0.62
50%	0.50	0.85	0.49	0.73	0.70	0.72
75%	0.64	0.88	0.65	0.88	0.80	0.80
Max	0.69	0.90	0.95	0.89	1.00	0.92

*Stats* statistical analysis, *std* standard deviation, *min* minimum, *max* maximum.

### Principal components analysis

**Figure 5:**
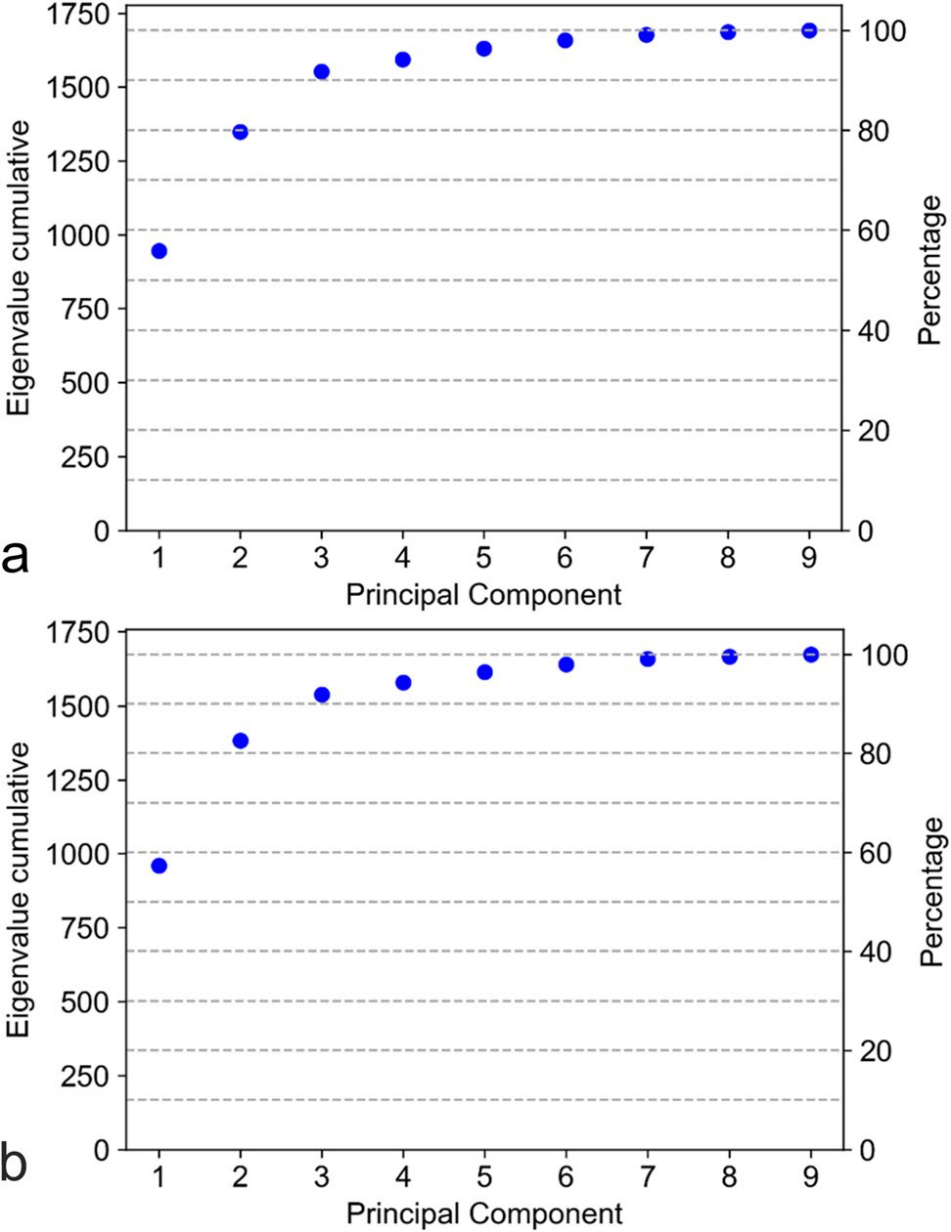
Principal component analysis (PCA) scree plots of retinal volumes S1–S9. Plots show the cumulative eigenvalues of the nine principal components (PCs) for (**a**) right and (**b**) left eyes. Eigenvalues indicate the explained variability of the respective PC. Additionally, the first three PCs explain 91.8% and 91.9% of the variability in the right and left eyes, respectively.

PCA yielded largely similar results in the right and left eyes (Fig. 5, Table 5). The first three principal components (PCs) explained 91.8% and 91.9% of the variability in the right and left eyes, respectively (Fig. 5). The first PC is a sort of average of the nine slice coefficients. The absolute values of the slice coefficients on the nasal-temporal axis are slightly larger than those on the superior-inferior axis (Table 5). Conversely, the second PC is a center-vs-edge factor on the superior-inferior axis (Table 5). It assigns relatively large positive and negative weights to the slices at the edges (S6, S8) and those near the center (S2, S4), respectively. The center slide (S1) received weights with small absolute values. The first two PCs are similar to those of a similar analysis in a previous choroid study^10^. Furthermore, the third PC is an inside-vs-outside factor on the nasal-temporal axis. In the right eye, it assigns relatively large negative and positive weights to the central slices (S1, S3, S5) and the peripheral slices (S7, S9), respectively. Moreover, in the left eye, the sign of the weights in the third PC was reversed with respect to the right eye.

**Table 5:** Coefficients of principal component analysis (PCA) for the first three principal components (PCs) for the right and left eyes.

PC	Eye	S1	S2	S3	S4	S5	S6	S7	S8	S9
1	Right	9.17	9.41	12.2	9.27	12.08	7.35	12.11	8.17	11.21
2	Right	− 1.11	− 9.19	1.88	− 8.81	1.62	10.93	− 0.94	10.46	− 1.66
3	Right	− 9.25	0.81	− 4.38	2.12	− 4.42	2.58	4.92	1.75	6.39
1	Left	9.85	8.99	12.41	8.93	12.37	7.14	12.02	8.04	11.66
2	Left	− 1.4	− 9.45	2.14	− 9.27	1.57	11.06	− 1.01	10.56	− 1.39
3	Left	8.59	− 0.58	3.06	− 1.51	3.36	− 1.8	− 5.02	− 1.13	− 5.42

*PC* principal component.

The table shows the PCA coefficients for the right (first three rows) and left (last three rows) eyes. The patterns are mostly similar for the right and left eyes. The first PC is a kind of average of the nine slices, with coefficients of the slices on the nasal-temporal axis slightly greater than that on the superior-inferior axis. The second PC mainly affects the superior-inferior axis, assigning relatively large positive and negative weights to the slices at the edges (S6, S8) and those near the center (S2, S4), respectively. The third PC is an inside-vs-outside factor on the nasal-temporal axis assigning relatively large negative and positive weights to the central slices (S1, S3, S5) and the peripheral slices (S7, S9), respectively, in the right eyes (sing is reversed in left eyes).

### Statistical hypothesis tests

#### Multivariate analysis of variance results

MANOVA was performed to investigate the effects of sex, origin, and their interactions on the nine slice coefficients (S1–S9). Because the nine slice coefficients are correlated (see Table 4), performing a joint analysis, which treats them simultaneously as dependent variables, is reasonable. MANOVA was performed separately for the right and left eyes. Notably, interactions between sex and origin were insignificant (significance level of 0.01) and thus were removed from the models. Moreover, sex and origin significantly affected the right and left eyes (Table 6).

**Table 6:** Multivariate analysis of variance (MANOVA) results. MANOVA was performed separately for the right (first two rows) and left eyes (last two rows).

Eye	Variable	Wilk’s lambda	Pr > F
Right	Sex	0.6812	8.5e−11
Right	Origin	0.6842	1.2e−10
Left	Sex	0.6905	3.4e−10
Left	Origin	0.6702	3.5e−11

Both origin and sex significantly affect the dependent variables S1–S9 in the right and left eyes (at significance level of 0.01). The effect size is measured using Wilks’ lambda. The test results are equivalent to the Pillai’s trace, Hotelling–Lawley trace, and Roy’s greatest root.

#### Analysis of variance results

MANOVA revealed that both sex and origin had a significant effect on the retinal volumes of slices S1–S9. Therefore, ANOVA was performed on individual slices to better understand the influence of sex and origin. The analysis was performed individually for each slice (S1–S9), considering sex, origin, and their interactions as independent variables. We note that the obtained *p*-values might not be completely accurate because the nine slice coefficients were correlated. Nevertheless, we considered the individual ANOVA to understand better which areas of the retina are influenced by sex and origin.

Rows one to five of Table 7 summarize the results of the statistical significance tests based on ANOVA regarding sex and origin. Additionally, their interactions were insignificant (significance level of 0.01/9) and were thus removed from the models. The results for the right and left eyes largely corresponded with each other. Sex significantly affected retinal volumes in S2, S4, S7, and S9. The origin had a significant effect on S1, S4, and S5. For ease of comparison, rows six to nine of Table 7 contain the results of the statistical hypothesis tests based on an equivalent ANOVA performed on the choroid volumes^11^.

**Table 7:** Summary of *p*-values in the analysis of variance (ANOVA) for measured retinal and choroidal slice volumes.

Variable	Layer	Eye	S1	S2	S3	S4	S5	S6	S7	S8	S9
Sex	Retina	Right		5.8e−9***		6.7e−13***			3.7e−5***		3.7e−7***
Origin	Retina	Right	9.8e−6***			1.6e−4**	3.0e−3*				
Sex	Retina	Left		1.5e−9***		7.4e−12***			4.0e−5***		8.6e−7***
Origin	Retina	Left	1.7e−5***			2.3e−3*	3.5e−3*				
Sex	Choroid	Right		2.3e−3*		1.4e−3*		2.0e−3*		1.5e−4**	
Origin	Choroid	Right									
Sex	Choroid	Left				2.9e−3*		1.3e−5***		1.3e−6***	
Origin	Choroid	Left									

*p* < 0.001/9: ***; *p* < 0.01/9: **; *p* < 0.05/9:*.

Three stars indicate *p*-values < 0.001/9. Two stars indicate *p*-values < 0.01/9. One star indicates *p*-values < 0.05/9. Nine is the number of hypotheses and, therefore, the factor applied to adjust significance levels in the Bonferroni correction. Exact *p*-values are only shown when the results are significant.

## Discussion

OCT has emerged as a generally used imaging technique that provides high-resolution cross-sectional images of various pathological findings in preclinical settings and enables reliable measurement of retinal thickness. Cynomolgus monkeys are ideal models for various congenital and acquired retinal diseases, such as retinitis pigmentosa^14^, glaucoma^9^, central serous chorioretinopathy^15,16^, and ocular safety studies. Notably, such data have paved the way for drug development, including photodynamic^17,18^ and anti-vascular endothelial growth factor therapies^19,20^. However, natural background variation, disparities in scan placement at consecutive imaging timepoints, and subjective semiquantitative assessments may cause iatrogenic variation and impact readout parameters^10,13^. A previous study identified origin and sex differences in cynomolgus monkey macular thickness^10^. However, these measurements were based solely on two-dimensional data; therefore, this study further defines the three-dimensional foveolar realm using automatic algorithms. An enormous sample size of 374 healthy foveal volumetric OCT images was recorded and was derived from 203 animals of Mauritian or Asian origin.

The central foveolar zone within 1 mm had a mean retinal volume of 0.205 mm^3^ (range 0.154–0.268 mm^3^). Interestingly, the overall variation of the retinal volume in this central area was relatively small (7.9% CV), and the retina was thinnest in the foveal subfield, as reported in other reports^21^.

Furthermore, sex and origin significantly influenced the foveolar-volume readings, as previously suggested in two-dimensional cynomolgus monkey foveolar OCT data^10^ and that of humans^22–24^.

In the choroid layer, in contrast, only sex had a significant influence on choroidal volumes but not origin^11^. Interestingly, the effect of sex appears to be different in the choroid and in the retina. Sex significantly affects volumes S2, S4, S6, and S8 in the choroid. But in the retina, it is volumes S2, S4, S7, and S9 that are significantly affected by sex (Table 7). These results could indicate a sexual dimorphism between male and female individuals that is differentially expressed in the retina and choroid.

This study had some limitations. First, OCT is based only on the reflectivity of the scanning laser signal. Therefore, not all layers may be equally detectable^3,25^, which can lead to falsely high or low retinal volume values. Additionally, data from different devices cannot be compared with each other because device-dependent differences have been found^26–29^. However, this was not the aim of this initial study, but it can be evaluated in more detail in the future.

## Conclusions

In summary, the central retinal volume showed a relatively low degree of variation. Nevertheless, there are significant and indeed natural appearing differences in retinal volume depending on the origin and sex of the animal, which should be taken into account when selecting animals for preclinical studies. In particular, for consecutive studies involving the same molecule, the geographical origin of the animals should be maintained.

## Supplementary Information

Supplementary Information.

## Data Availability

All relevant data are presented within this paper and its supporting information. All further information can be obtained on request from the corresponding author.
